# Learning Wireless Sensor Networks for Source Localization

**DOI:** 10.3390/s19030635

**Published:** 2019-02-02

**Authors:** S. Hamed Javadi, Hossein Moosaei, Domenico Ciuonzo

**Affiliations:** 1 Department of Electrical Engineering, Faculty of Engineering, University of Bojnord, Bojnord 9453155111, Iran; 2 Department of Mathematics, Faculty of Science, University of Bojnord, Bojnord 9453155111, Iran; hmoosaei@gmail.com; 3 Department of Electrical Engineering and Information Technologies, University of Napoli “Federico II”, 80100 Naples, Italy; domenico.ciuonzo@gmail.com

**Keywords:** Internet of Things, quadratic programming, region of event detection, source localization, support vector machine (SVM), target tracking, twin support vector machine (TWSVM), wireless sensor network (WSN)

## Abstract

Source localization and target tracking are among the most challenging problems in wireless sensor networks (WSN). Most of the state-of-the-art solutions are complicated and do not meet the processing and memory limitations of the existing low-cost sensor nodes. In this paper, we propose computationally-cheap solutions based on the support vector machine (SVM) and twin SVM (TWSVM) learning algorithms in which network nodes firstly detect the desired signal. Then, the network is trained to specify the nodes in the vicinity of the source (or target); hence, the region of event is detected. Finally, the centroid of the event region is considered as an estimation of the source location. The efficiency of the proposed methods is shown by simulations.

## 1. Introduction

The applications of the networks of wireless smart motes with sensing, processing and communication capabilities are expanding. Among all the possible applications, these networks are applicable for preventing people from suffering health effects such as sleep disturbance [1,2], annoyance [3], cardiovascular effects [4], learning impairment [5,6], and hypertension ischemic heart disease [7]. Other examples include noise monitoring [8] for obtaining noise maps and related action plans [9].

These wireless sensor networks (WSN) are the basis of the emerging technology of Internet of Things (IoT) and specifically fit very well in surveillance applications [10,11], where detecting an event source in a region of interest (ROI) and tracking it are important challenges.

Tracking over WSNs is a challenging problem since it needs implementing statistical filters with cumbersome computations. These computationally heavy algorithms should be run by each network node with severe limitations of processing power, memory, and communication.

The nodes’ estimations of the source track are transmitted to a central unit of network which is referred to as the “fusion center (FC)”. FC adopts an appropriate fusion rule for obtaining a tuned estimation of the track. Some of the existing fusion rules are Independent likelihood pool (ILP) [12], Covariance intersection (CI) fusion [13], Information graph [14], Track-to-track fusion [15], and Consensus-based fusion (distributed MTT–DMTT) [16].

There are also source localization approaches based on information theoretic methods. In the approach presented in [17], a coarse estimation of the source location is obtained based on the data of specified nodes which are assigned as the anchor nodes. Then, a set of non-anchor nodes with maximum mutual information (MI) between the source location and their measurements are activated. The source is localized after several iterations. The complexity of the MI-based method grows exponentially in the network size. To alleviate this problem, another sensor selection scheme for source localization based on conditional posterior Cramer-Rao Lower Bound (PCRLB) has been proposed by [18,19].

In many applications, it is important to detect the region of event after detection of its occurrence. In other words, many applications need to divide ROI into event and non-event regions after the event occurrence is detected. For example, in fire detection in forests or poisonous gas leakage applications, it is crucial to detect the region of event during time in order to estimate the event expansion rate and schedule appropriate plans.

Due to severe bandwidth and energy limitations of battery-powered sensor nodes, they are usually programmed to send as little data as possible. The extreme case in event detection is implementation of distributed detection [20] where each node sends just one bit to FC indicating its decision about event occurrence. Apparently, the detection performance of nodes is not perfect and includes false alarms and misses of the event occurrence. However, if erroneous decisions of nodes can be corrected, the region of nodes with decision ‘1’ (indicating event occurrence) would imply the event region. The goal of this paper is to correct both false alarms and misses of the network nodes using appropriate machine learning (ML) methods in order to estimate both event location and its region.

In any WSN, we encounter distributed agents gathering data for a specific task. Since the size of data collected by WSNs is usually enormous, the related problems and challenges can be handled by suitable ML methods. Instances of applying ML methods on different aspects of WSNs—such as node localization, channel allocation, and routing—have been reviewed in [21].

Support vector machines (SVMs) are powerful tools for classification and regression [22,23,24] which are based on the statistical learning theory. Compared with other ML methods such as artificial neural networks, SVMs have a better generalization guarantee. SVM divides the data samples of two classes by determining a specific hyperplane in the input space. This hyperplane maximizes the separation between the two classes by solving a quadratic programming problem (QPP) whose solution is globally optimum.

Inspired by the idea of SVM, twin support vector machine (TWSVM) for classification problems was proposed in [25]. TWSVM seeks two non-parallel proximal hyperplanes for classifying data sets. Unlike SVM, TWSVM solves two small SVM-type problems; i.e., TWSVM solves a pair of QPPs instead of a single complex QPP as in SVM. SVM and TWSVM also work effectively for classifying data sets which are not linearly separable by using the theory of kernel functions [26].

In this paper, two source localization methods are proposed based on distributed detection together with SVM and TWSVM algorithms. Our contributions presented in this paper are as follows:

- Distributed detection is implemented over a WSN in order to alleviate both computational and communication burdens (the two most important limitations of WSNs);
- By applying the SVM and TWSVM learning methods, the network becomes capable of detecting the nodes in vicinity of the desired event by whom the region of the event is detected;
- The event location is assumed to be the centroid of the event region. However, a correction method is provided for cases wherein the event occured at the edges of ROI;
- We show that our proposed methods—referred to as “Red-S” and “Red-T”, respectively, since they perform the region of event detection (Red) by applying SVM and TWSVM, respectively—are not only good at source localization but also are capable of tracking a moving source.

The proposed algorithms are more general and practical than the existing learning methods such as the one in [27] where the region of event is detected via data exchange among neighbor nodes and the false alarm probability is restrictively assumed the same as the miss probability.

The remaining of this paper is organized as follows. The models and assumptions used throughout this manuscript are discussed in Section 2. Section 3 provides the required background knowledge. Estimation of the event location and region of event detection by using SVM and TWSVM are proposed in Section 4. The evaluation results is discussed in Section 5. Finally, the paper is concluded in Section 6.

*Notations*: Lower-case (resp. upper-case) bold letters denote vectors (resp. matrices), with $a_{i}$ (resp. $A_{i,j}$) representing the *i*th element (resp. (i,j)th element) of a (resp. A). In addition, I is used for denoting the identity matrix, 1 is a vector of all elements 1 with proper size, $a^{T}$ (resp. $A^{T}$) denotes the transpose of a (resp. A), the 2-norm of vector a will be denoted by ∥a∥, and N(μ,Σ) denotes a normal distribution with mean vector μ and covariance matrix Σ. In addition, U(a,b) indicates the uniform distribution between *a* and *b*. Finally, the symbol ∼ means “distributed as”.

## 2. System Model and Assumptions

**Figure 1:**
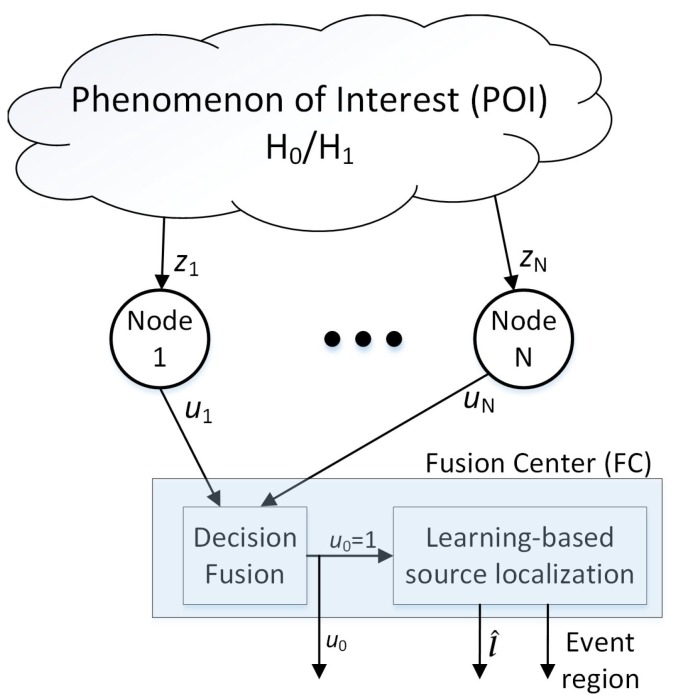
The network configuration studied in this paper. The network nodes observe a common phenomenon of interest and decide locally about event occurrence. Then, they send their decisions to FC where the final decision $u_{0}$ is taken. If $u_{0}=1$, FC gives an estimation of source location $\hat{l}$ and its region.

The system model shown in Figure 1 is adopted throughout this paper. Different parts of this model are discussed in the following subsections.

### 2.1. Node Model

A network of *N* random-deployed wireless smart motes (referred to as “nodes”) is considered, programmed for collaborative detection of a desired event occurrence. In fact, there are two conditions: the normal condition in which no event has occurred (denoted by hypothesis $H_{0}$), and event occurrence (denoted by hypothesis $H_{1}$). Node *i*, $i\in \left\{1,\ldots ,N\right\}$ observes a common phenomenon of interest (POI) for detection of a desired scalar parameter $\theta \left(l\right)$ originating from the event location l according to the observation model:

(1) $$z_{i}=\left\{\begin{array}{c} g\left(\theta \left(l\right)\right)+v_{i},\;H_{1}\\ v_{i},\;H_{0} \end{array}\right.$$

where $v_{i}$ is a zero-mean additive white Gaussian noise (AWGN) with variance $\sigma^{2}$ (i.e., $v_{i}∼N\left(0,\sigma^{2}\right)$), and g(.) is a mapping function (e.g., a function indicating the attenuation of an acoustic signal originating from position l). It is assumed that the noises of nodes, $v_{i}$, are uncorrelated both temporally and spatially.

Each node *i* takes a local decision $u_{i}$ about event occurrence based on an optimal decision rule—later discussed in Section 3.3—and sends it to an FC (Figure 1). Here, note that the scalar observation model (1) has been adopted just for simplicity in our discussions. If the desired parameter (signal) is multi-dimensional, just the decision rule would simply change. Nevertheless, we adopt the scalar observation model since detection is not the focus of our study.

### 2.2. Network Model

We denote the location of node i,i∈{1,…,N} by $x_{i}$ and collect all locations in $x=\left\{x_{1},\ldots ,x_{N}\right\}$. In order to detect the event region, it is assumed that the nodes’ locations are known by FC. The positions may be obtained by using an appropriate localization method such as those presented in [28,29].

As illustrated in Figure 1, a parallel configuration has been adopted in this paper. The parallel configuration is the most popular configuration in the literature. However, nodes may transmit their decisions to FC indirectly through intermediate nodes in a hop-by-hop manner (since their communication range is usually limited). This configuration can be represented by the parallel configuration as well if the communication channels are assumed to be error-free [30,31,32].

### 2.3. Communication Channel Model

The communication channels between nodes and FC are assumed to be ideal and error-free. In practice, there are two sources of error: (i) erroneous local decisions, which are due to either local false alarms or misses of nodes, and (ii) erroneous received decisions due to the faulty nature of wireless channels. We integrate both error sources into just the first type and ignore the communication error rate since it does not affect the implementation and evaluation of our proposed algorithms at all.

### 2.4. Problem Statement

After the event occurrence is detected by FC, the problems are to:

1. estimate the event location l, and
2. divide the region into event and non-event regions.

## 3. Backgrounds

In this section, we review briefly the SVM (Section 3.1) and TWSVM (Section 3.2) learning algorithms. The section ends with a refresher of detection over WSN (Section 3.3), needed for the discussion of the proposed methods.

### 3.1. Support Vector Machine (SVM)

The support vector machines (SVMs) are a popular class of supervised learning algorithms. A comprehensive tutorial on the topic could be found in [33]. In its original formulation, SVM attempts to separate two classes of data by a hyperplane whose distance from the data of each class is maximized.

**Figure 2:**
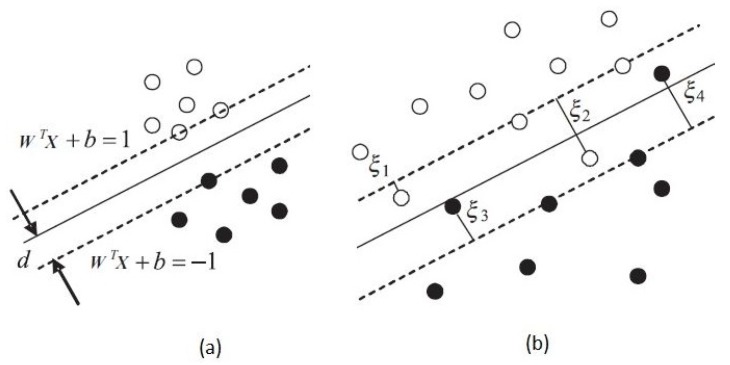
(**a**) Support Vector Machine (SVM) procedure for separating two classes of data by a hyperplane whose distance from the nearest samples of the classes (i.e., the margin *d*) is maximized. (**b**) SVM for the non-separable case in which the samples within the margin are penalized [33].

Let us denote the training set by $x=\{x_{1},\ldots ,x_{N}\}$ where the class of each sample $x_{i}$ is represented by $y_{i}\in \left\{-1,1\right\}$. The goal of SVM is to separate the two classes of data by a hyperplane $y=w^{T}x+b$ whose distance with the nearest samples is maximized. To that end, the following constrained optimization problem must be solved [33]:

(2) $$\begin{array}{c} \min_{w,b,\xi_{i}}\;\frac{1}{2}{∥w∥}^{2}+C\sum_{i=1}^{N}\xi_{i}\\ s.t.\;y_{i}\left(w^{T}x+1\right)\geq 1-\xi_{i}\\ \;\xi_{i}\geq 0 \end{array}$$

where $\left\{\xi_{i}\right\}$s are slack variables used for non-separable samples, and C>0 is a penalty term used for allowing some limited misclassifications. More specifically, as shown in Figure 2a, the SVM algorithm attempts to separate the two classes by maximizing the margin around the optimum hyperplane. However, there would be borderline samples in most practical cases that make classification challenging (Figure 2b). The slack variables $\xi_{i}$ are used for taking these borderline samples into account by imposing a penalty for them. $\xi_{i}=0$ for samples out of the margin, $0<\xi_{i}<1$ for samples within the margin, and $\xi_{i}>1$ for samples that are misclassified.

The optimization problem (2) can be solved by resorting to its dual problem and forming the Lagrange objective function as follows:

(3) $$\begin{array}{cc} \min_{\alpha } & \frac{1}{2}\alpha^{T}YRY\alpha -\alpha^{T}1\\ s.t. & \sum_{i=1}^{N}y_{i}\alpha_{i}=0\\ & 0\leq \alpha_{i}\leq C,\;i=1,\cdots ,N \end{array}$$

where Y is a diagonal matrix with $Y_{ii}=y_{i}$, α is the column vector of the Lagrange coefficients, 1 is an N×1 vector of all elements 1, and R is the matrix of the inner products of the samples with its elements given by:

(4) $$R_{i,j}=x_{i}^{T}x_{j}$$

Now, a quadratic optimization problem has been obtained that can be solved by using appropriate functions in related libraries of programming languages, such as the function quadprog(.) in MATLAB. After solving (3) for α, the solution to the optimum hyperplane is given by:

(5) $$\begin{array}{c} w=\sum_{i=1}^{N}\alpha_{i}y_{i}x_{i}\\ b=y_{i}-w^{T}x_{i} \end{array}$$

The final decision rule for each input vector x is now:

(6) $$\begin{array}{c} f\left(x\right)=\mathrm{sign}\left(\sum_{i=1}^{N}\alpha_{i}y_{i}x_{i}^{T}x+b\right) \end{array}$$

where sign(.) is the sign function.

Though the SVM algorithm discussed above is applicable just in cases in which the two classes can be separated by a linear hyperplane, it can be used in nonlinear cases via the Mercer kernels. Using Mercer kernels, the data is mapped into a higher dimensional space wherein the two classes are linearly separable. To that end, one should simply replace the inner product matrix R in (3) with an appropriate kernel matrix. As an instance, a popular kernel—also used in this paper—is the Gaussian kernel. Using the Gaussian kernel, the kernel matrix is built with its elements given by:

(7) $$K_{i,j}≜K\left(x_{i},x_{j}\right)=\exp\left(-\frac{{∥x_{i}-x_{j}∥}^{2}}{2\sigma^{2}}\right)$$

Then, the decision function (6) generalizes to:

(8) $$f\left(x\right)=\mathrm{sign}(\sum_{i=1}^{N}\alpha_{i}y_{i}K\left(x,x_{i}\right)+b)$$

### 3.2. Twin Support Vector Machine (TWSVM)

In this subsection, Twin Support Vector Machine (TWSVM) [25] is discussed as a popular and accurate method of data classification. TWSVM formulation is similar to that of SVM except that in TWSVM, all the data points do not emerge in the constraints, simultaneously. Furthermore, TWSVM is proved to be faster than SVM as it solves two smaller sized QPPs [25].

**Figure 3:**
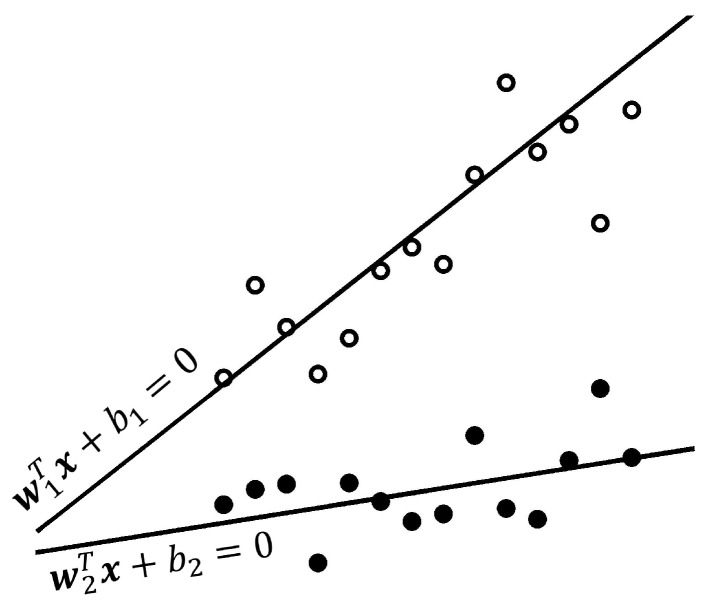
Geometric interpretation of Twin Support Vector Machine (TWSVM).

Denoting the data points in class +1 and class −1 by matrices $A\in R^{m_{1}\times n}$ and $B\in R^{m_{2}\times n}$, respectively, ($m_{1}+m_{2}=N$ and *n* is the size of each feature) with $A_{i}$ and $B_{i}\in R^{n}$ denoting the *i*th row of A and B, respectively. The linear TWSVM, unlike SVM, finds a pair of nonparallel hyperplanes as follows:

(9) $$\begin{array}{c} w_{1}^{T}x+b_{1}=0\;\mathrm{and}\;w_{2}^{T}x+b_{2}=0 \end{array}$$

where $w_{1},w_{2}\in R^{n}$, and $b_{1},b_{2}\in R$. Each hyperplane of TWSVM is close to the data points of one of the two classes while it is distant from the data points of the other class. Therefore, the formulation of TWSVM can be stated as follows:

(10) $$\begin{array}{cc} \min_{w_{1},b_{1},q_{1}} & ∥Aw_{1}+1b_{1}{∥}^{2}+c_{1}1^{T}q_{1}\\ s.t. & -\left(Bw_{1}+1b_{1}\right)+q_{1}\geq 1\\ & q_{1}\geq 0 \end{array}$$

(11) $$\begin{array}{cc} \min_{w_{2},b_{2},q_{2}} & ∥Bw_{2}+1b_{2}{∥}^{2}+c_{2}1^{T}q_{2}\\ s.t. & \left(Aw_{2}+1b_{2}\right)+q_{2}\geq 1\\ & q_{2}\geq 0 \end{array}$$

where $c_{1},c_{2}$ are positive parameters, and $q_{1},q_{2}$ are the vectors of the related slack variables. It is clear that the purpose of TWSVM is to solve two QPPs (10) and (11). Each QPP in the TWSVM pair represents a classic SVM formulation, with the exception that not all data points show up in the constraints of either problem (see Figure 3).

The corresponding Wolfe dual problems can be obtained respectively as follows:

(12) $$\begin{array}{cc} \max_{\alpha } & 1^{T}\alpha -\frac{1}{2}\alpha^{T}G{\left(H^{T}H\right)}^{-1}G^{T}\alpha \\ s.t. & 0\leq \alpha_{i}\leq c_{1},i\in \left\{1,\ldots ,m_{2}\right\} \end{array}$$

(13) $$\begin{array}{cc} \max_{\gamma } & 1^{T}\gamma -\frac{1}{2}\gamma^{T}H{\left(G^{T}G\right)}^{-1}H^{T}\gamma \\ s.t. & 0\leq \gamma_{i}\leq c_{2},i\in \left\{1,\ldots ,m_{1}\right\} \end{array}$$

where α and γ are Lagrange multipliers, H≜[A1], and G≜[B1].

It can be shown that the solutions to (12) and (13) are given by ${\left[w_{1}\;\;b_{1}\right]}^{T}=-{\left(H^{T}H\right)}^{-1}G^{T}\alpha$ and ${\left[w_{2}\;\;b_{2}\right]}^{T}={\left(G^{T}G\right)}^{-1}H^{T}\gamma$.

The matrices $H^{T}H$ and $G^{T}G$ are positive semidefinite, thus they may be singular. Therefore, in order to avoid ill-conditioned cases, the inverse matrices ${\left(G^{T}G\right)}^{-1}$ and ${\left(H^{T}H\right)}^{-1}$ are approximately replaced by ${\left(G^{T}G+\delta I\right)}^{-1}$ and ${\left(H^{T}H+\delta I\right)}^{-1}$ respectively, where δ is a very small positive scalar.

The nonlinear TWSVM can be expressed as follows:

(14) $$\begin{array}{cc} \min_{w_{1},b_{1},q_{1}} & ∥K\left(A,C^{T}\right)w_{1}+1b_{1}{∥}^{2}+c_{1}1^{T}q_{1}\\ s.t. & -\left(K\left(B,C^{T}\right)w_{1}+1b_{1}\right)+q_{1}\geq 1\\ & q_{1}\geq 0 \end{array}$$

(15) $$\begin{array}{cc} \min_{w_{2},b_{2},q_{2}} & ∥K\left(B,C^{T}\right)w_{2}+1b_{2}{∥}^{2}+c_{2}1^{T}q_{2}\\ s.t. & \left(K\left(A,C^{T}\right)w_{2}+1b_{2}\right)+q_{2}\geq 1\\ & q_{2}\geq 0 \end{array}$$

where $C^{T}≜{\left[A\;\;B\right]}^{T}$, and K(.,.) is the kernel matrix of an appropriately chosen type (the Gaussian kernel in (7) here for its elements). The Wolfe dual problems of (14) and (15) to obtain the following hypersurfaces

(16) $$\begin{array}{c} K\left(x^{T},C^{T}\right)w_{1}+b_{1}=0\;\;\mathrm{and}\;K\left(x^{T},C^{T}\right)w_{2}+b_{2}=0 \end{array}$$

are respectively as follows:

(17) $$\begin{array}{cc} \max_{\alpha } & 1^{T}\alpha -\frac{1}{2}\alpha^{T}G^{\prime }{\left({H^{\prime }}^{T}H^{\prime }\right)}^{-1}{G^{\prime }}^{T}\alpha \\ \overset{˚}{s.t.} & 0\leq \alpha_{i}\leq c_{1},i\in \left\{1,\ldots ,m_{2}\right\} \end{array}$$

(18) $$\begin{array}{cc} \max_{\gamma } & 1^{T}\gamma -\frac{1}{2}\gamma^{T}H^{\prime }{\left({G^{\prime }}^{T}G^{\prime }\right)}^{-1}{H^{\prime }}^{T}\gamma \\ s.t. & 0\leq \gamma_{i}\leq c_{2},i\in \left\{1,\ldots ,m_{1}\right\} \end{array}$$

where $H^{\prime }≜\left[K\left(A,C^{T}\right)\;\;\;1\right]$ and $G^{\prime }≜\left[K\left(B,C^{T}\right)\;\;\;1\right]$. Solving the above problems gives ${\left[w_{1}\;\;b_{1}\right]}^{T}=-{\left({H^{\prime }}^{T}H^{\prime }+\delta I\right)}^{-1}{G^{\prime }}^{T}\alpha$, and ${\left[w_{2}\;\;b_{2}\right]}^{T}={\left({G^{\prime }}^{T}G^{\prime }+\delta I\right)}^{-1}{H^{\prime }}^{T}\gamma$. Then, the distance of each input data to each of the two obtained hypersurfaces determines its class. In other words, for each input data x, its class *y* is determined by:

(19) $$\begin{array}{c} y=2\left(\arg\min_{j\in \{1,2\}}\left|w_{j}^{T}x+b_{j}\right|-1.5\right),\;i\in \left\{1,\ldots ,N\right\} \end{array}$$

### 3.3. Detection over Sensor Networks

In this subsection, the theory of detection and its implementation over sensor networks are reviewed. A comprehensive tutorial on the topic is [20].

Detection is a basic task of WSNs in many applications [34,35] where a final decision about either an event occurrence (labeled as “hypothesis $H_{1}$”) or no event occurrence (denoted by “hypothesis $H_{0}$”) must be taken. This final decision is taken by FC via fusing the data of the network nodes.

The network nodes may send either raw or processed observations to FC. While sending raw measurements imposes a considerable communication burden on the network, the nodes are usually programmed to make a local decision and inform FC about their decision by sending just one bit. To have this practically popular scheme—known as “distributed detection”—implemented, two types of decision rules must be designed: a local decision rule for each node, and a decision fusion rule for FC. These two kinds of decision rules are discussed in what follows.

#### 3.3.1. Local Decision Rule

Nodes of the network observe a desired signal θ according to model (1). In practical scenarios, there is no information about the specifications of the source signal (e.g., the location and the strength of the acoustic signal are not known). In these cases, it is proved that the optimal decision rule for each node would be the likelihood ratio test (LRT) if the statistical information of the sensing noise ($v_{i}$ in (1)) is available [36]. Accordingly, the optimal decision rule for node *i* with observation model (1) is given by [36]:

(20) $$u_{i}=\left\{\begin{array}{c} 0,\;z_{i}^{2}<\tau_{i}\\ 1,\;z_{i}^{2}>\tau_{i} \end{array}\right.$$

with $u_{i}=0$ ($u_{i}=1$) indicating $H_{0}$ ($H_{1}$), and $\tau_{i}$ being the detection threshold that is given based on a local false alarm rate (i.e., $\mathrm{Pr}\left(u_{i}=1|H_{0}\right)$).

#### 3.3.2. Fusion Rule

The network nodes send their decision to FC where a decision fusion rule should be adopted in order to have a final decision. One simple and popular one is the counting rule [37] in which the sum of received decisions is simply compared against a threshold:

(21) $$\Lambda ≜\sum_{i=1}^{N}u_{i}\overset{H_{1}}{\underset{H_{0}}{≷}}t$$

where *N* indicates the network size, and *t* is the detection threshold which is obtained according to a desired network false alarm rate. Under a common threshold for sensors and the same noise statistics (i.e., in homogeneous WSNs), Λ under $H_{0}$ (i.e., $\Lambda |H_{0}$) is binomial distributed while it follows a Poisson-binomial distribution in more general cases [38]. The threshold *t* is computed according to this distribution. While simple, the counting rule maintains robustness [39,40] and can reach almost the optimum detection performance in large network sizes [20] (i.e., in sufficiently large values of *N*). There are modifications of the counting rule such as LVDF [41] and WDF [35,42]. Each node in LVDF modifies its decision based on the majority of the decisions of its neighbors. In WDF, the decision of each node is weighted based on the node’s sensed signal-to-noise ratio (SNR) and then the weighted decisions are counted. Moreover, the network’s detection performance can be improved by considering the correlation among the nodes’ decisions [43,44].

## 4. Learning Methods for Source Localization

In this section, we propose two learning-based methods for both source localization and obtaining the nodes affected by the event. The complexity of the methods and their designing methodology are discussed as well.

### 4.1. Region of Event Detection by SVM (Red-S)

In this section, our first proposed method of source localization in WSNs is presented. Having it implemented in a WSN, we show that the region of event can be recognized. This method is referred to as “*Red-S*” which is carried out by the following steps:

1. The network nodes observe ROI in order to detect a desired event (e.g., a desired target) when it occurs by using the decision rule (20). Then, they send their decisions to an FC where the final decision is taken by exploiting an appropriate fusion rule such as the counting rule (21). It is assumed that the network size is sufficiently large so that the overall detection performance of the network is optimum (i.e., there is neither a false alarm nor a miss).
2. Upon event detection, FC runs the SVM algorithm in order to detect the region of the event (i.e., the nodes in vicinity of the event) as follows:
  (a) The locations of the nodes are considered as the training set $x=\left\{x_{1},\ldots ,x_{N}\right\}$ with $x_{i}$ being the location of node *i*.
  (b) The decision of each node denotes its class according to the following mapping rule:
    (22) $$\begin{array}{c} u_{i}=0\to y_{i}=-1\\ u_{i}=1\to y_{i}=1 \end{array}$$
    where i∈{1,…,N}.
  (c) The Gaussian kernel (7) is adopted for constituting the kernel matrix K that replaces the matrix R in the optimization problem (3). Designing parameter σ of the Gaussian kernel is elaborated in Section 4.3.1.
  (d) Having SVM solved, the coefficient vector α is obtained based on which the classifier parameters w and *b* are obtained.
  (e) The event region is obtained by applying the nodes’ locations to the obtained classifier as follows:
    (23) $$y=\mathrm{sign}\left(KY\alpha +b1\right)$$
    where $y_{i}=1$ denotes that node *i* is in the even region while it is not if $y_{i}=-1$. Note that the above relation is the vectorized version of (8).
3. The location of the event is estimated by averaging the locations of the nodes in the region of the event. In other words, the centroid of the nodes in the vicinity of the event is considered as the location of the event. More specifically, denoting the set of the nodes in event region by $E≜\left\{x_{i}:y_{i}=1,i=1,\ldots ,N\right\}$, the estimation of the event location is given by:
  (24) $$\hat{l}=\frac{1}{\left|E\right|}\sum_{i\in E}x_{i}$$
  where |E| denotes the cardinality of E.

In summary, the locations of nodes are used as the training set together with the nodes’ decisions as their classes. After having the network trained, the nodes’ locations are applied to the obtained classifier. The result gives the region of event with its centroid as the estimation of the event’s location.

### 4.2. Region of Event Detection by TWSVM (Red-T)

Red-S gives both event location and its region after the event occurrence is detected by FC. To improve the accuracy of classification of ROI into event and non-event regions, the more advanced TWSVM algorithm is applied. However, TWSVM is more sensitive to design parameters—as discussed later—and needs a priori estimation of event location in order to yield accurate outcomes. This estimation can be provided by Red-S. In other words, the classification performance of Red-S is improved by applying TWSVM on the network. The overall algorithm is referred to as “Red-T” and performs the following steps after Red-S result is out:

1. The parameters of TWSVM are computed based on the classification result of Red-S as will be elaborated in Section 4.3.2.
2. The TWSVM classification algorithm with its parameters computed in the previous step is applied on the nodes’ locations as the training set. The class of each node is determined according to the mapping rule given in (22).
3. TWSVM gives two hypersurfaces based on the training set by solving (17) and (18).
4. The nodes are assigned to regions of event and non-event depending on to which of the two hypersurfaces given by (17) and (18) are closer. In other words, the class of node *i* is updated according to:
  (25) $$\begin{array}{c} y_{i}=2\left(\arg\min_{j\in \{1,2\}}\left|w_{j}^{T}x_{i}+b_{j}\right|-1.5\right),\;i\in \left\{1,\ldots ,N\right\} \end{array}$$
  where $y_{i}=1$ denotes that node *i* is in the region of event while it is not in the region of event if $y_{i}=-1$.
5. Similarly as in Res-S, the location of event is obtained by averaging all nodes in event region, i.e., by (24).

**Figure 4:**
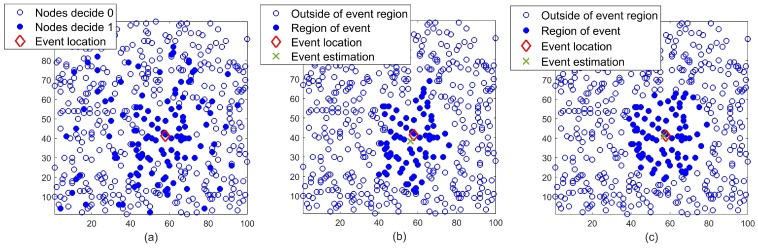
(**a**) Distributed detection in a wireless sensor network with 500 nodes randomly deployed in a 100 m × 100 m region and local false alarm probability $p_{fa}=0.1$. (**b**) Event localization after applying Red-S. (**c**) Event localization after applying Red-T.

As an instance of how Red-S and Red-T work, see Figure 4 where the classification results of applying Red-S and Red-T to the network condition in Figure 4a has been illustrated in Figure 4b,c, respectively. As shown, both event location and its region have been detected in both methods; though the performance has been improved by applying Red-T. Note that the nodes with false alarms are ignored by Red-S and Red-T while the decisions of the nodes which are in the vicinity of the event but miss the detection are corrected.

### 4.3. Designing Parameters

#### 4.3.1. Red-S Parameters

During implementation of Red-S, the value of σ in the Gaussian kernel (7) is of importance. Its value should be chosen so that a reasonable kernel value is obtained. Therefore, it can be considered as a function of the average distance between nodes, or simply a function of network density. For example, the average distance between nodes is less in dense networks, so σ should be adjusted to be less as well.

#### 4.3.2. Red-T Parameters

The performance of TWSVM depends on the choices of the kernel function parameters. The optimal values for the parameters are determined by the grid search method with the aim of minimizing the distance between the estimated location of event by TWSVM and that of Red-S.

To alleviate the computational burden, the settings $c_{1}=c_{3}$ and $c_{2}=c_{4}$ in (17) and (18) are used. As in [45], the grid values for $c_{1},c_{2},c_{3}$, and $c_{4}$ are considered to be in $\left\{10^{i}|i=-10,-4,\ldots ,10\right\}$ and the kernel width σ chosen from the range $\left\{10^{i}|i=-5,-4,\ldots ,1\right\}$ for each dataset.

### 4.4. Edge Effect Correction

As discussed in previous sections, the centroid of the nodes in the event region, i.e., (24), is considered as the source location. Since the centroid is never located in the edges of the region under surveillance (ROI), Red-S and Red-T do not perform well at the edges of ROI; we refer to this fault as the *edge effect*.

The edge effect is alleviated by adding (or subtracting) a random number to the already obtained estimation of the source location. More specifically, if a portion of the nodes in event region, say 20 percent of them, are among the nodes with the lowest 2nd coordination (e.g., the nodes of the left edge of the network shown in Figure 1), the event may have been occurred at the edge. Thus the first coordination of $\hat{l}$ should be corrected by:

(26) $$\hat{l}\left(1\right):=\hat{l}\left(1\right)-2u$$

where := indicates modification (like assignment operator in programming languages), and *u* is a uniformly distributed random variable between 0 and $\hat{l}\left(1\right)$, i.e., $u∼U(0,\hat{l}\left(1\right))$. The same correction mechanism may be applied for other edges. Such correction is validated in Section 5.

### 4.5. Computational Complexity

SVM requires to solve a QPP with inequality constraints. It is well-known that for a training data set of size *N*, the computational complexity of SVM is $O\left(N^{3}\right)$ [46,47]. On the other hand, as discussed in Section 3.2, TWSVM solves a pair of QPPs instead of a single complex QPP of SVM. If the number of patterns in each class approximately equals to N/2, the complexity of TWSVM is $O\left(2{\left(N/2\right)}^{3}\right)$ [25]. In other words, TWSVM is four times faster than SVM. Accordingly, the complexities of Red-S (involving SVM) and Red-T (involving both SVM and TWSVM) are $O\left(N^{3}\right)$ and $O\left(\frac{5}{4}N^{3}\right)$, respectively.

Note that both outstanding source localization methods based on statistical signal processing, MI-based and PCRLB-based methods, are iterative. Moreover, the nodes quantize their observations into *L* bits and *A* nodes are selected in each iteration [18]. The complexity of MI-based method in *each iteration* is $O\left(NL^{A}+ALN\right)$ [18] which grows exponentially with the network size (more precisely, with the number of nodes selected in each iteration). The PCRLB is much simpler with its complexity being $O\left(ALN\right)$ [18] in each iteration. Therefore, Red-S and Red-T, while consuming much less bandwidth with sending just one bit by each node, provide complexity reduction, especially compared to the MI-based method.

## 5. Evaluation and Discussion

In this section, the performance of the Red-S and Red-T schemes is evaluated. To that end, a randomly deployed homogeneous WSN in a 100 m × 100 m region is considered where nodes’ observations are contaminated by a standard normal AWGN. It is assumed that the network goal is to detect a target with an acoustic signal with the following isotropic model:

(27) $$P_{i}=\frac{P_{0}}{1+d_{ti}^{a}}$$

where $P_{i}$ denotes the strength of the acoustic source at the location of node *i*, $P_{0}$ is the strength of the source in 1 m distance from the target, $d_{ti}$ is the distance between node *i* and the target, and *a* is an attenuation coefficient (a=2 is considered throughout our simulations). Therefore, the observation of node *i* can be modeled as:

(28) $$z_{i}=\sqrt{P_{i}}+v$$

In simulations, C=0.5 has been used as the trade-off parameter of the SVM optimization problem, and $\sigma =\frac{20000}{N}$ as the kernel parameter (i.e., σ=20 is used for N=1000). The accuracy of Red-S and Red-T for source localization has been evaluated in terms of mean squared error (MSE) defined by:

(29) $$MSE=E\left({∥l-\hat{l}∥}^{2}\right)$$

in which l and $\hat{l}$ are respectively the exact and the estimated location of the target, and E(.) denotes the statistical expectation.

The proposed methods are compared against the fault recognition (FR) algorithm [27]. In FR, the potentially faulty decision (i.e., either false alarm or miss of the event) of each node is meant to be corrected by comparing it with the decisions of the node’s neighbors. In fact, FR exploits the spacial correlation among nodes’ decisions and works as follows:

1. Determine the neighbors of each node (i.e., the nodes in the communication range of each node).
2. Nodes take an initial decision based on their observations.
3. The final decision of each node is equal to the decision of majority of its neighbors.

**Figure 5:**
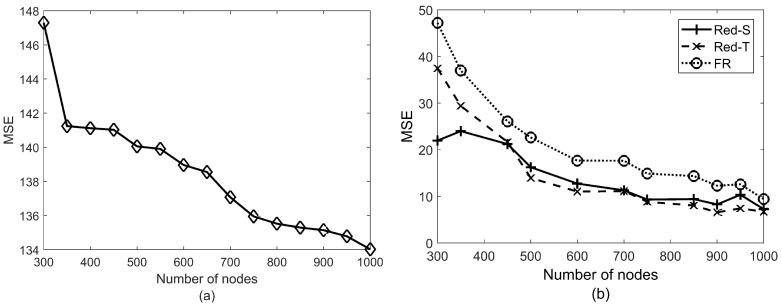
(**a**) Mean squared error (MSE) of naive source localization. (**b**) MSE of Red-S, Red-T, and FR in different number of nodes (*N*). $P_{0}=1000$ and the local false alarm probability $p_{fa}=0.1$ have been considered in simulations.

**Figure 6:**
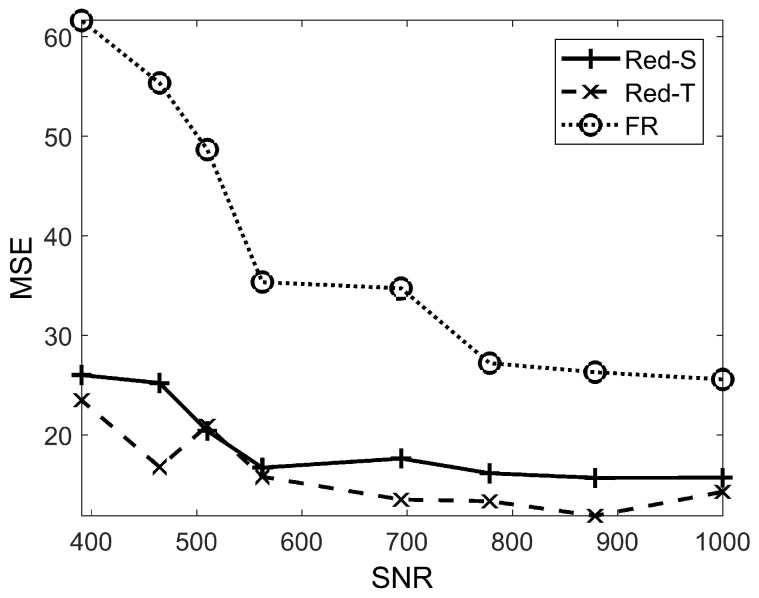
Mean squared error (MSE) of Red-S, Red-T, and FR in different values of signal-to-noise ratio (SNR) in a 500-node network. The local false alarm probability $p_{fa}=0.1$ has been considered in simulations.

**Figure 7:**
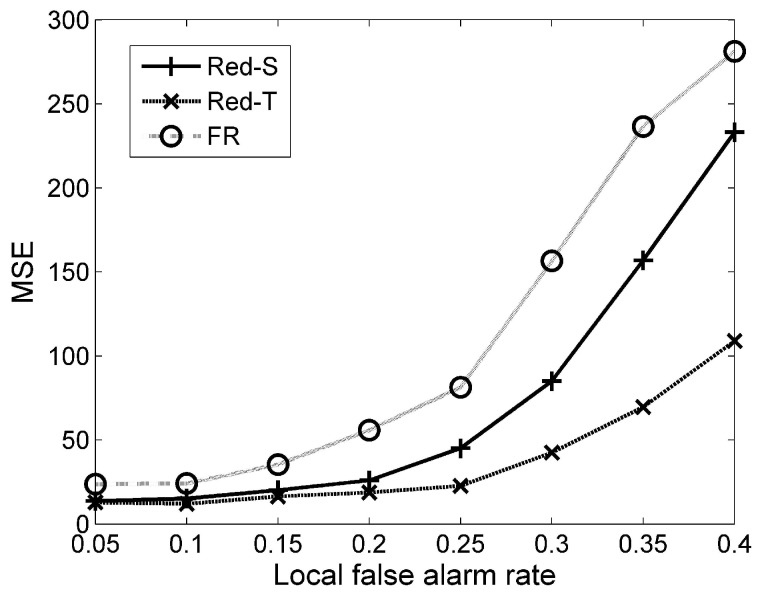
Mean squared error (MSE) of Red-S, Red-T, and FR vs. the false alarm probability of local nodes in a 500-node network. $P_{0}=1000$ has been considered in simulations.

The MSEs of the methods have been obtained via more than 5000 Monte-Carlo runs in different scenarios whose results have been depicted in Figure 5, Figure 6 and Figure 7. In addition to FR, we also examined the naive source localization where simply the centroid of all nodes with $u_{i}=1$ is considered as the estimation of the source location. However, the MSEs of this method were more than three times those of FR (more than 120 m${}^{2}$ in all cases). Therefore, its performance is included just in Figure 5 where the MSEs of the methods are shown in different network sizes. Comparison of the performance of naive source localization in different network densities (Figure 5a) with that of the other three methods (Figure 5b) reveals how much the performance is improved by processing the raw decisions.

Figure 5b shows that the estimation error decreases in more dense networks. However, the decrease rate becomes negligible after a specified density. In other words, it is not advantageous to employ more network nodes after a specified density. For example, in our scenario in Figure 5b, the difference between the MSE of a 600-node and a 1000-node networks is less than 4 m${}^{2}$. Figure 5b also shows that MSE of Red-S yields a more accurate estimation of the event location in a less dense network. This is because the parameters of Red-T are more sensitive to the dynamics of networks. In addition, note that the grid search of the TWSVM algorithm has been confined in order to speed up the running time. In fact, Red-S is more robust than Red-T because there is just one parameter to be adjusted.

It has been shown in Figure 6 that the estimation error is lower in more values of SNR. In addition, Figure 7 shows how local false alarm rates affect the overall target tracking error. Note that nodes send more data (and hence consume more energy) in more false alarm rates. Therefore, the false alarm rate must be as low as possible. However, the very low local false alarm rate decreases the detection probability of the network as well. Therefore, there is a trade-off here.

**Figure 8:**
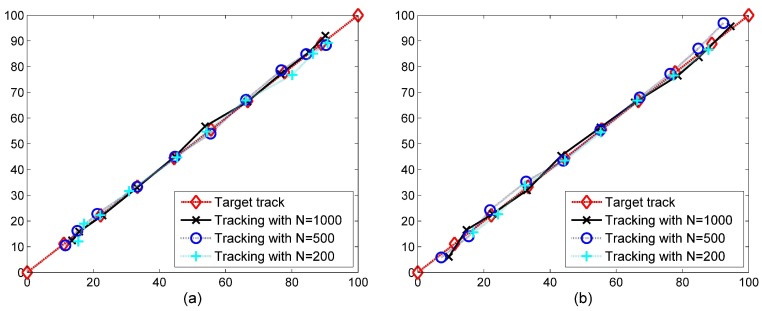
Accuracy of Red-S in tracking a moving target through a network in a 100 m × 100 m region. *N* indicates the number of network nodes. $P_{0}=1000$ and local false alarm rate $p_{fa}$ have been considered in the simulations. (**a**) Without edge effect correction. (**b**) With edge effect correction.

In another scenario, shown in Figure 8, the accuracy of Red-S in tracking a moving target has been assessed. In this scenario, a target moves from the lowest left corner to the highest right corner in a constant speed. Figure 8 shows the average of 10 tracking performances. As is shown, the tracking performance is better in more dense networks. In addition, note that the accuracy is acceptable in even networks with low densities. Another point is that the performance is not appropriate in corners since the centroid of the region of the event is considered as the location of the event in Red-S which is never located at the edges of ROI. However, as shown in Figure 8b, the edge effect is alleviated appropriately, especially in denser cases, by applying the correction method discussed in Section 4.4.

## 6. Conclusions and Future Directions

In this paper, two methods of source localization were proposed for implementing in a WSN with low-cost nodes. In our proposed methods, the network firstly detects the region of the desired event and then computes the event location by averaging the locations of all nodes in the vicinity of the event. To reach that, a combination of distributed detection and learning algorithms is exploited. In fact, the source localization is carried out in three phases: (i) Network nodes send their decisions about event occurrence to FC. (ii) Upon event detection, the FC classifies the nodes into two classes of nodes in “event region” and nodes in “non-event region”. (iii) The average of the locations of the nodes in the region of the event is considered as the event location.

The region of the event detection is specified by using SVM and TWSVM learning methods. Therefore, the proposed methods were referred to as “Red-S” and “Red-T”, respectively. By simulating different scenarios, it was shown that Red-S and Red-T work appropriately in target localization, especially in dense networks. However, the proposed methods are capable of localizing just one event. Applying learning methods, such as the *k*-mean clustering method, for multi-source localization may be considered as a future work.

Finally, note that though the proposed methods were applied on the WSN framework, they are applicable to every framework with false alarms and misses. One such example is medical health tests. The methods can also be applied on outlier detection applications.

## Abbreviations

The following abbreviations are used in this manuscript:

WSN	Wireless Sensor Network
IoT	Internet of Things
ML	Machine Learning
QPP	Quadratic Programming Problem
SVM	Support Vector Machine
TWSVM	Twin SVM
FC	Fusion Center
AWGN	Additive White Gaussian Noise
SNR	Signal-to-noise ratio
FR	Faulty Recognition algorithm
Red-S	Region event detection using SVM
Red-T	Region event detection using TWSVM
ROI	Region of Interest
